# Delayed and progressive damages to juvenile mice after moderate traumatic brain injury

**DOI:** 10.1038/s41598-018-25475-9

**Published:** 2018-05-09

**Authors:** Shu Zhao, Xiaoting Wang, Xiang Gao, Jinhui Chen

**Affiliations:** Spinal Cord and Brain Injury Research Group, Stark Neuroscience Research Institute, Department of Neurosurgery, Indiana University, 320W 15th street, Indianapolis, IN 46202 United States

## Abstract

Symptoms are commonly more severe in pediatric traumatic brain injury (TBI) patients than in young adult TBI patients. To understand the mechanism, juvenile mice received a controlled cortical impact (CCI) injury at moderate level. Tissue lesion and cell death were measured and compared to our previous reports on brain injury in the young adult mice that received same level of impact using same injury device. Tissue lesion and cell death in the cortex was much less in the juvenile mouse brain in the first few hours after injury. However, once the injury occurred, it developed more rapidly, lasted much longer, and eventually led to exaggerated cell death and a 32.7% larger tissue lesion cavity in the cortex of juvenile mouse brain than of young adult mouse brain. Moreover, we found significant cell death in the thalamus of juvenile brains at 72 h, which was not commonly seen in the young adult mice. In summary, cell death in juvenile mice was delayed, lasted longer, and finally resulted in more severe brain injury than in the young adult mice. The results suggest that pediatric TBI patients may have a longer therapeutic window, but they also need longer intensive clinical care after injury.

## Introduction

Traumatic brain injury (TBI) is a leading cause of death and disability in children^1^. This age group also has the most increase each year in the rate of TBI-related emergency department (ED) visits occurring in the past 10 years. According to the Centers for Disease Control and Prevention (CDC), in 2012, about 329,290 children were treated at EDs in U.S^2^. The cost of pediatric TBI was more than 1 billion dollars^3^, imposing a huge financial burden to families. Moreover, the poor outcome of pediatric TBI affects not only the patients, but also their whole family for an indefinite time.

Unlike TBI in an adult, an injury to a child’s brain affects an organ that is still developing. The brain is not fully grown at birth, not only in terms of its size, but also in terms of the complexity of the neural networks that determine how it functions^4,5^. The functional impact of TBI in children can be different than in adults. Studies of brain tissues from toddler TBI patients also reported dramatic damage following injury^6,7^. Younger ages showing less resilience to the injury with more profound long-term deficits in cognition and sociability^8^. Children TBI is associated with persistent negative effects on psychosocial development including academic struggles as well as an inability to develop adequate social skills^9^. Pediatric patients are also more likely to exhibit long-term symptoms during development, such as depression, anxiety, learning deficits, and attention deficits, among others^10–15^. These deficits occurring after TBI resulted in poorer social, emotional, and sociosexual outcomes^16,17^, and a tendency towards increased aggression, which evolves across phases of development to adulthood^18^. The full sequelae of pediatric TBI can emerge and/or persist well into adulthood, leading to the perspective that TBI in children is a chronic disease process rather than a one-time event^19–21^.

Pediatric TBI has attracted more and more attention in the basic research. It has been reported that TBI in the immature mouse or rat models that use CCI or fluid percussion injury showed progressive neuronal loss^8,22–26^, impaired axonal transport and neurofilament compaction^27^, and axonal degeneration^28–31^. TBI in immature rodents also compromised blood brain barrier permeability and the inflammation response^32^. David Hovda’s research with rats also found that it took immature animals with a mild head injury 6 to 10 times longer to recover than mature rats with the same type of injury^33^. These studies showed that TBI cause dramatic injury to immature brain. However, the mechanisms influencing the vulnerability of the young brain to TBI are poorly understood. To study pediatric TBI we chose mice at the postnatal age of 21 days, which corresponds to human toddlers at the age of 2–3 years^18,34,35^. According to CDC, children from 0–4 years old showed the highest rate of emergency department visitation. As well, children of pre-school age, especially toddlers aged 2–3 years, showed worse outcomes after injury than older children^36^, thus we focused our study on this age group. The model chosen to induce TBI was the controlled cortical impact (CCI) model, a widely used model for TBI. The same parameter was chosen to induce CCI injury in young adult mice as we previously described^37,38^. After injury, histology and staining for cell death were applied to assess the brain damage after CCI injury on juvenile brains. The results were then compared to our previous reports on brain injury in the young adult mice.

## Materials and Methods

### Animal care

Mice (C57/BL6) were housed with a 12/12 light/dark cycle and were given food and water ad libitum according to the principles outlined in “Guidelines for Care and Use of Experimental Animals.” All the mice were used in experiments at the age of 21 days postnatal, which corresponds to human toddlers at the age of 2–3 years. All procedures were performed under protocols approved by the Animal Care and Use Committee at the Indiana University. All experiments were performed in accordance with guidelines and regulations of Indiana University Biosafety Committee.

### Controlled Cortical Injury model

Mice at 21 days old received a moderate CCI injury or a sham operation following the procedures we previously reported^37–51^. Briefly the mice were anesthetized with Avertin and placed in a stereotaxic frame (Kopf Instruments, Tujunga, CA) for craniotomy. The scalp was pulled and the skull was exposed. The surgical field was 4 mm in diameter. The center of the surgical field was identified midway between the lambda and bregma sutures, and midway between the central suture and the temporalis muscle laterally. Then the skullcap was carefully removed without disruption of the underlying dura. The impacting piston was angled to make the impacting tip (3 mm in diameter) perpendicular to the exposed cortical surface. Mice received the impact according to parameters we described before: the amount of deformation was set at 1.0 mm and the piston velocity controlled at 3.0 m/sec. These modifications resulted in a moderate level of injury using an electromagnetic model (Impact One TM Stereotaxic Impactor for CCI, Leica Microsystem, and Illinois USA). Sham (non-injured) animals received the craniotomy, but no CCI injury.

### Tissue preparation

All animals were deeply anesthetized with Avertin and then perfused transcardially with cold saline, followed by a fixative containing 4% paraformaldehyde (PFA) in PBS. The brains were removed, post-fixed overnight in PFA, and cryoprotected for 48 hours in 30% sucrose. Serial 30 μm thick coronal sections were cut using a cryostat (Leica CM 1950), and stored at −20 °C. The sections were then processed for histopathological analysis.

### Nissl staining

Nissl staining was performed to analyze anatomical structure changes. Briefly, sections were incubated in a solution of 0.1% cresyl violet (Sigma) for 30 min. After a quick rinse in distilled water, the sections were then differentiated in 95% ethanol for 5 min and followed by dehydration in 100% ethanol for 10 min, 2 times. The sections were then cleared in xylene for 5 min, 2 times. After they were air-dried, the sections were mounted with DPX (Sigma).

### Cortical Tissue Lesion Cavity Measurement

Series of every 1 in 6 sections (30 μm thickness, 180 μm apart) from covered injured cortex were stained with cresyl violet to show the spare cortex. The boundary contours of the contralateral and ipsilateral spare cortex were drawn with a Zeiss microscope attached to a Neurolucida system (Microbrightfield Inc., Colchester, VT). The enclosed volume within the contours was measured. The percent cortex of the cavity was calculated with the following formula:

$$\begin{array}{rcl}{\rm{percentage}}\,{\rm{of}}\,{\rm{the}}\,{\rm{cortical}}\,{\rm{cavity}} & = & ({\rm{contralateral}}\,{\rm{cortex}}\,{\rm{volume}}\\  &  & -\,{\rm{ipsilateral}}\,{\rm{spare}}\,{\rm{cortex}}\,{\rm{volume}})\\  &  & /{\rm{contralateral}}\,{\rm{cortex}}\,{\rm{volume}}\times 100 \% .\end{array}$$

### Fluoro-Jade B staining

Sections were stained with Fluoro-Jade B (FJB) to detect neuronal death. Briefly, sections were hydrated in distilled water for 5 min. The sections were then incubated in a solution of 0.06% potassium permanganate (Sigma) for 20 min at room temperature. After they were washed in distilled water for 5 min, 2 times, the sections were incubated in a 0.0004% solution of FJB (Sigma) for 20 min, followed by washing in distilled water 3 times and incubation in a solution of 0.01% 4′,6-diamidino-2-phenylindole (DAPI) (Sigma) for 10 min. Sections were then air-dried and mounted with DPX.

### Neuronal Cell Death Counting in the Cortex Surrounding the Contusion Site

The total number of FJB-positive neurons in the damaged cortical area surrounding the impact area following TBI was determined through a blinded quantitative histological analysis (5 mice for each time point) following the protocol with modification^52,53^. Three sections per mouse in each group were chosen based on their close proximity to the epicenter of the impact, and the density of FJB-positive neurons within the damaged area near the impact site was quantified. Though rare, regions of tissue that were already dead and no longer had any positive signals were not included in the area/volume measured. Using Stereo Investigator software (MicroBrightfield Inc., Williston, VT), the tissue boundaries were defined and traced at 5× magnification on an Axio Imager M2 microscope (Zeiss). Counting of FJB-positive neurons was performed using a systematic sampling site method of the selected tissue area at 40× magnification for accurate recognition. The size for each site was set at 300 × 300 µm^2^ and the counting frame used was 100 × 100 µm^2^. FJB-positive neurons were selected based on morphology and fluorescent signal strength. After both the estimated population of FJB-positive neurons and specified tissue volume (in cubic millimeters [mm^3^]) were obtained, the density of cells per cubic micrometer was found using the following formula:

$$\begin{array}{rcl}{\rm{cell}}\,{\rm{density}} & = & ({\rm{estimated}}\,{\rm{cell}}\,{\rm{population}})/({\rm{tissue}}\,{\rm{volume}}\,{\rm{in}}\,{{\rm{mm}}}^{3}\\  &  & \times 1000000000\,\mu {{\rm{m}}}^{3}/{{\rm{mm}}}^{3})\end{array}$$

### Neuronal Cell Death Counting in the Hippocampus

Consistent with our previous report^43^, the total number of FJB-positive neurons in the hippocampus following TBI was determined through a blinded quantitative histological analysis (5 mice for each time point). After sectioning, 1 in 6 sections of the entire extent of the hippocampal formation was selected for assessment with FJB staining. Every FJB-positive neuron was counted and was determined under a fluorescent microscope with a 20× objective. Finally, the total number of FJB-positive neurons on any given section was calculated.

### Statistical analysis

All data were presented as average ± SD and analyzed using One-way ANOVA followed by LSD post hoc test. The significant level was set as *p* < 0.05.

## Results

### Moderate TBI induced progressive tissue lesion in the cortex of juvenile mice

**Figure 1:**
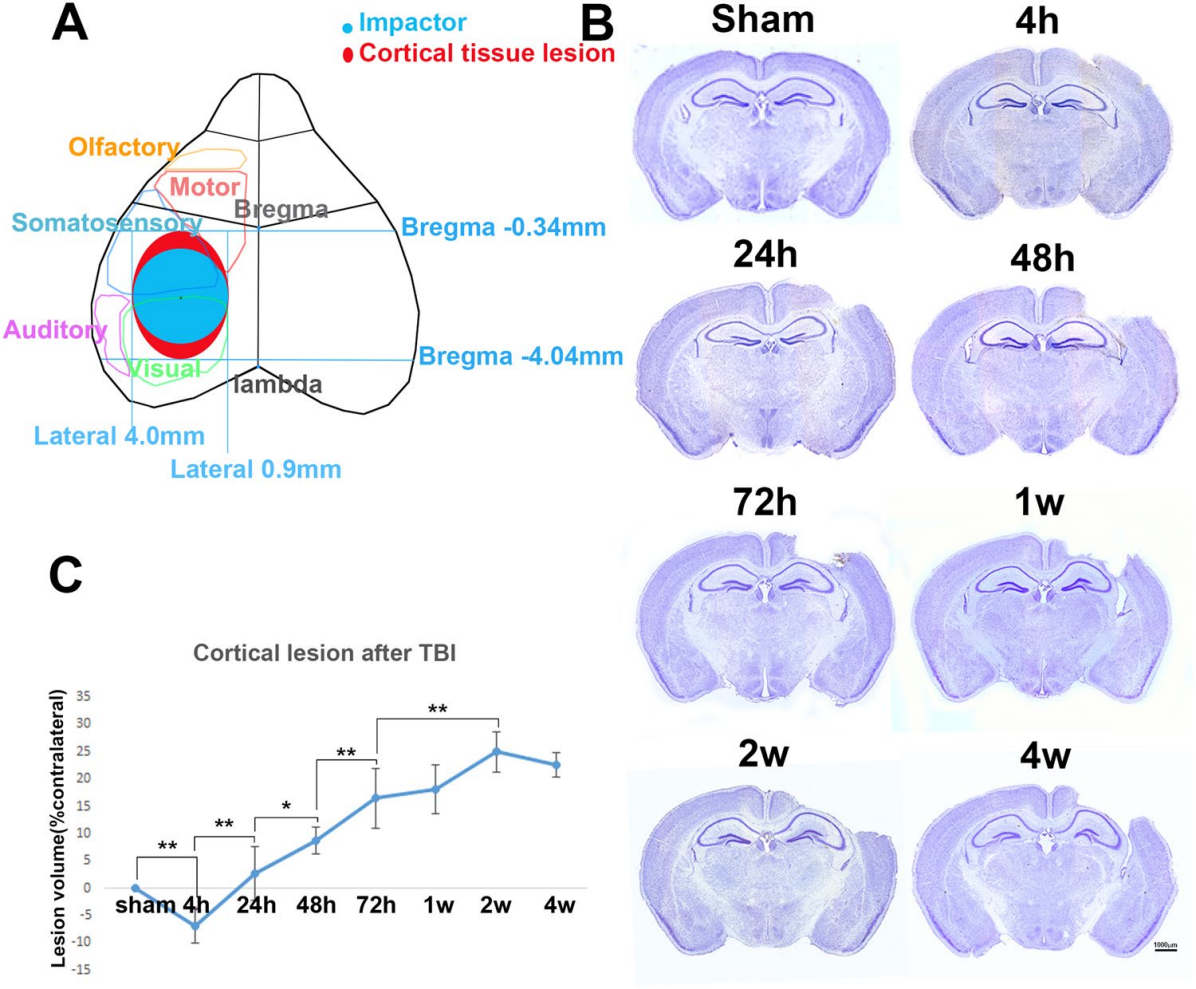
TBI caused an expanding cavity in juvenile cortex. (**A**) The illustration of the cortical tissue lesion after CCI surgery. (**B**) Nissl staining showed the temporal pattern of anatomical structure change. (**C**) Quantification of the cortical cavity (n = 4, *p < 0.05; **p < 0.01).

Postnatal mice at the age of 21 days were considered as corresponding to 2–3 year old human toddlers^18,34,35^. Juvenile mice at this age do not depend on their mother’s milk, and have learned independent feeding habits and survival strategies in their environment. The sizes of their cortexes also have reached adult size (Supplemental Figure 1). Thus we used the juvenile mice at this age as a model to study TBI in toddlers. Juvenile male mice at the age of 21 days received either a moderate CCI injury or a sham surgery with same level of impact using same injury device as in our previous studies in young adult mice^37,38,42,45,51^. The mice were sacrificed at 4 h, 24 h, 48 h, 72 h, 1 wk, 2 wk, or 4 wk after surgery (n = 4 at each time point). The tissue that made direct contact with the actuator is indicated in light blue (Fig. 1A).

Brains were removed and series of brain sections (1 out of 6) were selected to assess the injury in the brain using Nissl staining to reveal their gross anatomical structures (Fig. 1). The contour of the cerebral cortex of both ipsilateral brain and contralateral brain were outlined and their volumes were calculated using a *Neurolucida* system. The tissue lesion volumes were then calculated following the formula described in the Materials and Methods section and were converted to cortical lesion volumes (Fig. 1C). Very little tissue lesion was observed in the cortex at 4 h after TBI (Fig. 1B). The cavity in the cortex became obvious at 24 h after injury (Fig. 1B). The cavity in the cortex continued to increasingly enlarge after 24 h and up to 2 wk (Fig. 1B). A top view of the tissue lesion in the cortex, indicated in red (Fig. 1A), was located mainly between bregma −0.34 mm to bregma −4.04 mm, and medial 0.9 mm to lateral 4.0 mm. Most of the lost tissue was the sensory cortex above the hippocampus, and only a little bit was motor cortex (Fig. 1A).

Quantitative analysis showed that the cortex cavity volume was −6.92 ± 3.16% at 4 h after injury. The negative number likely resulted from an increase of cortex volume due to edema that is greater than the cortex lesion volume due to injury. Calculations of the cortex cavity volume showed a negative value at this time point, which is not observed in the adult brain following TBI. This result may suggest a dramatic occurrence of edema in the juvenile mouse brain, and may be greater than in the young adult mouse brain following TBI. From post-injury 4 h to 24 h, the size of cavity sharply changed from a negative number to 2.74 ± 4.89% (*p* = 0.001); from 24 h to 48 h, the cavity enlarged by 6% to 8.77 ± 2.39% (*p*_(24h vs. 48h)_ = 0.029); from 48 h to 72 h, the cavity enlarged by 8% to 16.47 ± 5.47%, (*p*_(48h vs. 72h)_ = 0.007). The size of the cavity continued to enlarge 72 h after injury, reaching 18.09 ± 4.44% at 1 wk, and reaching 24.98 ± 3.71% at 2 wk (*p*_(72h vs. 2w)_ = 0.003).

Two weeks after injury, the cavity had not enlarged further, but reduced slightly to 22.63 ± 2.26%, which may have been due to tissue deformation. The difference in cavity size between 2 wk and 4 wk was not statistically significant (*p*_(2w vs. 4w)_ = 0.376) (Fig. 1C). These data suggest that the brain tissue became dramatically edematous at least in the first few hours after injury; significant tissue lesion in the cortex did not occur until 4 h later; the tissue lesion became dramatically larger from 4 h after injury and continued enlarging for at least 2 wk.

### TBI caused dramatic neuronal death in the cortex of juvenile mice after injury

**Figure 2:**
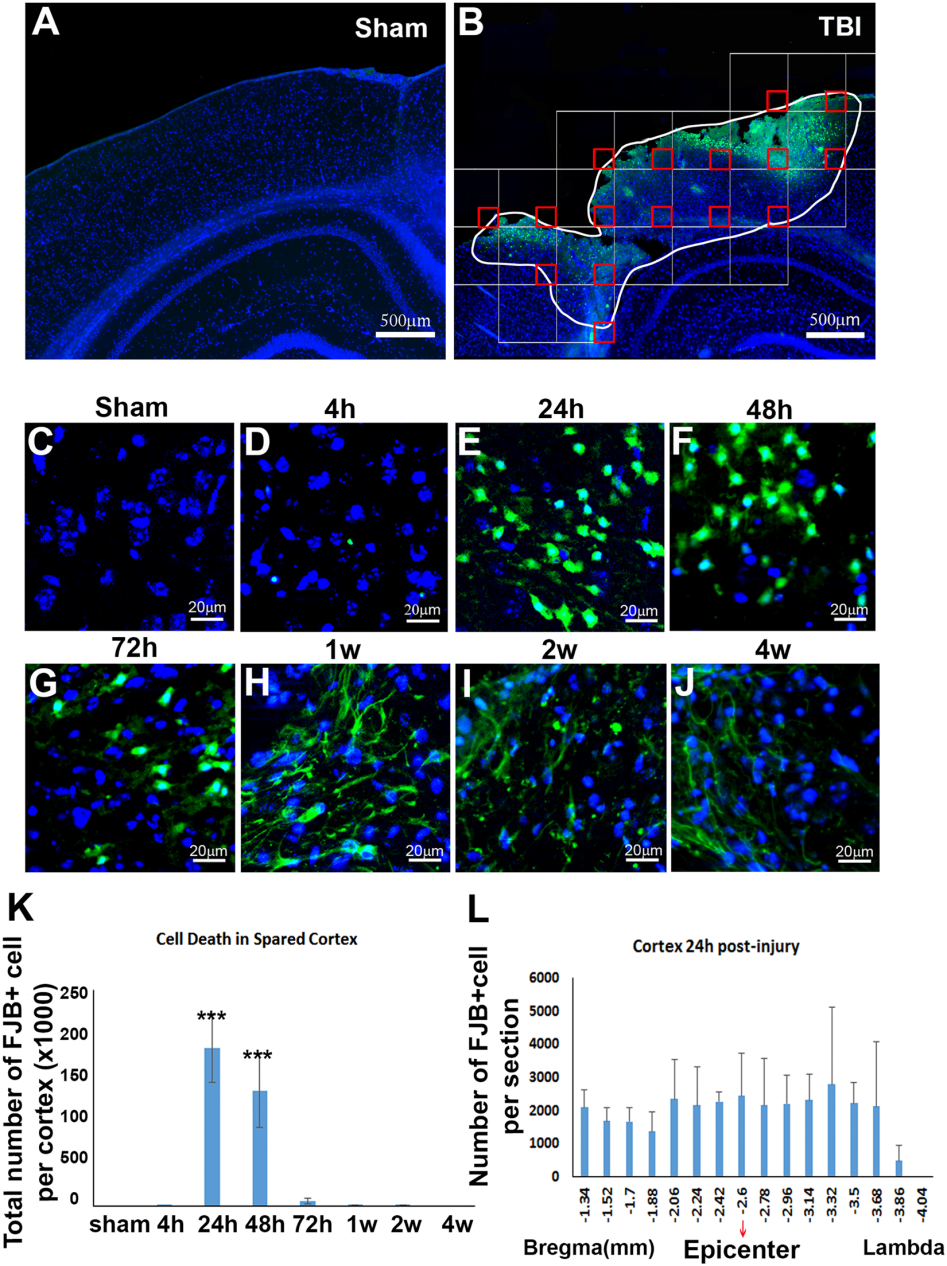
Temporal and spatial patterns of neuronal death in spared cortex after TBI. (**A**) Cortex with sham surgery stained with Fluoro-Jade B. (**B**) Fluoro-Jade B staining (green) showed the neuronal death in cortex after injury and stereological counting. (**C**–**J**) The temporal pattern of neuronal death in cortex after sham surgery or injury. (**K**) Quantification of FJB stained cells in cortex (n = 4, ***p < 0.001). (**L**) The rostral-caudal distribution of FJB-positive cells in spared cortex at post-injury 24 h.

To further assess brain damage in the juvenile cortex after CCI injury, we evaluated neuronal death in the cortex at different times after injury. Series of brain sections (1 out of 6) from the study above were stained with Fluoro-Jade B (FJB), which is a marker widely used to detect neuronal death in the central nervous system after injury^40,43,47,54^. The FJB-positive cells were observed only in the ipsilateral cortex (Fig. 2B), while no FJB-positive cells were observed in the cortex of sham-surgery mice (Fig. 2A). FJB-positive cells were rarely observed at 4 h after injury. The number of FJB-positive cells dramatically increased to 182130 ± 39745 cells/ipsilateral cortex at 24 h after injury (*p* = 0.000, Fig. 2B), and then decreased by 26.7% to 133242 ± 43148 cells/ipsilateral cortex at 48 h post-injury (*p* = 0.003). At 72 h after injury, the FJB-positive cells further sharply reduced by 95% to 5532 ± 3413 cells/ipsilateral cortex (*p* = 0.000, Fig. 2K). From then to the end of our observations, only a very small number of FJB-positive cells were observed. At 1 wk, 336 ± 252 cells/ipsilateral cortex were counted; at 2 wk, 225 ± 84 cells/ipsilateral cortex were counted; and, at 4 wk after injury, FJB-positive cells were rarely observed. In summary, a large number of neurons were induced to death in the ipsilateral cortex of juvenile mice, and >99% of cell deaths occurred between 24 h to 48 h after injury.

When we further determined the distribution of FJB-positive cells in the cortex of juvenile mice at 24 h after injury, we found FJB-positive cells widely spread along the rostral-caudal axis from bregma −1.34 mm to bregma −3.86 mm, while along the medial-lateral axis from medial 1.09 mm to lateral 4.12 mm (Fig. 2L).

### TBI induced delayed and dramatic neuronal death in the hippocampus of juvenile mice after injury

**Figure 3:**
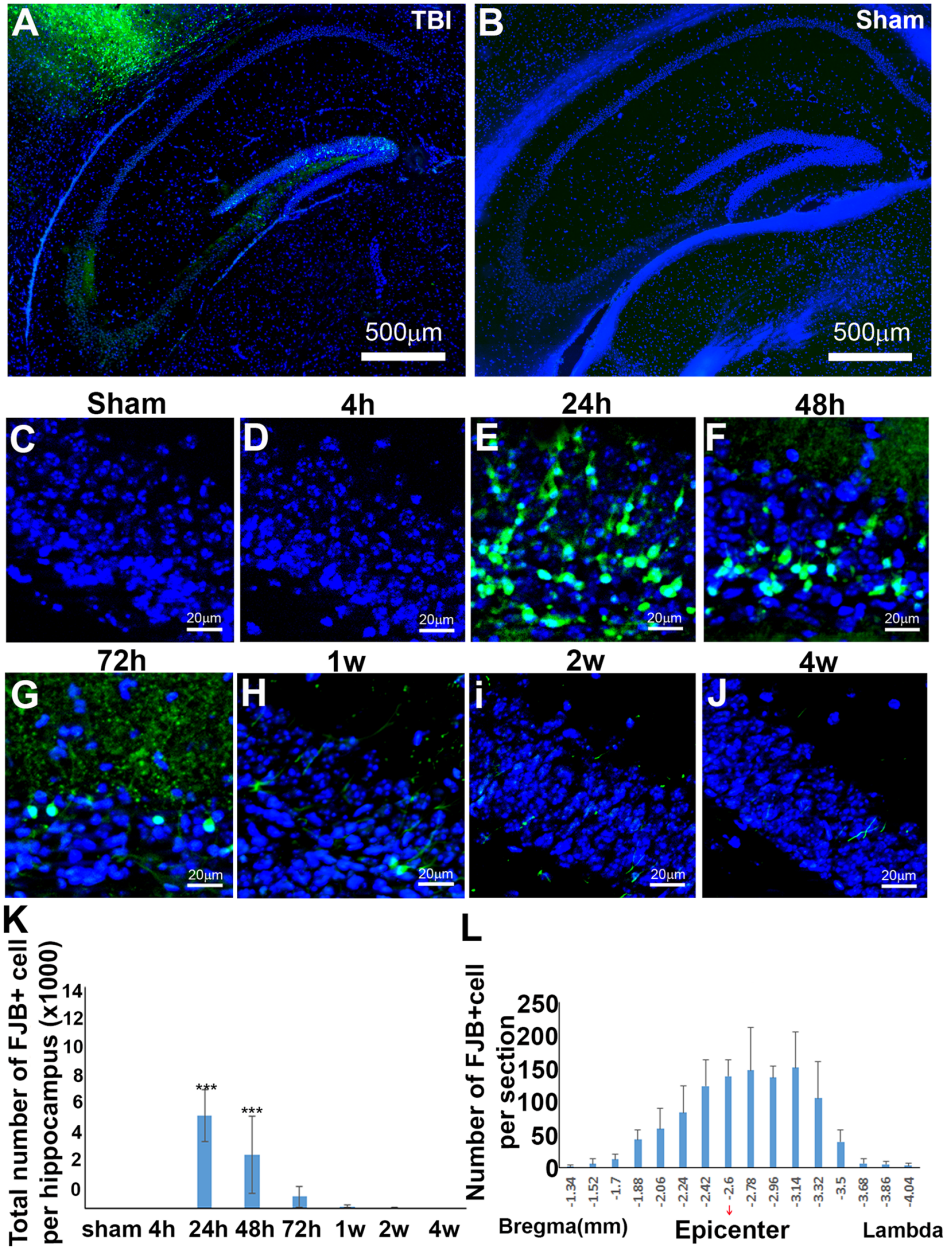
Temporal and spatial patterns of neuronal death in hippocampus after TBI. (**A**) Fluoro-Jade B staining (green) showed the neuronal death in hippocampus after injury. (**B**) Fluoro-Jade B the hippocampus of mouse received sham surgery. (**C**–**J**) The temporal pattern of neuronal death in hippocampus after sham surgery or injury. (**K**) Quantification of FJB stained cells in hippocampus (n = 4, ***p < 0.001) (**L**) The rostral-caudal distribution of FJB-positive cells in ipsilateral hippocampus at post-injury 24 h.

Similar to what we reported before^43,51^, TBI not only induced neuronal death in the cortex, it also significantly caused neuronal death in the hippocampus. Thus we further assessed the cell death in the hippocampus at 4 h, 24 h, 48 h, 72 h, 1 wk, 2 wk and 4 wk after injury. Brain sections containing hippocampus were selected to stain with FJB and the FJB-positive cells were counted. FJB-positive cells were observed in the ipsilateral hippocampus (Fig. 3A), but FJB was not detected in the contralateral hippocampus of injured mice or in the hippocampi of sham-surgery mice (Fig. 3B). FJB-positive cells were rarely identified in the ipsilateral hippocampus of juvenile mice at 4 h after injury. Similar to what we observed in the cortex, the number of FJB-positive cells sharply increased to 6318 ± 1757 cells/ipsilateral hippocampus (*p*_(4h vs. 24h)_ = 0.000) (Fig. 3K). The number of FJB stained cells then decreased by 43% to 3608 ± 2619 cells/ipsilateral hippocampus at 48 h post-injury (*p*_(24h vs. 48h)_ = 0.004), and further reduced 78% from 48 h to 72 h, reaching 795 ± 738 cells/ipsilateral hippocampus at 72 h after injury (*p*_(48h vs. 72h)_ = 0.002). Similarly to what we observed in cortex, from 72 h post-injury, the number of FJB-positive cells in hippocampus of juvenile mice also decreased dramatically. At 1 wk after injury, 164 ± 90 FJB-positive cells/ipsilateral hippocampus were counted; at 2 wks after injury, 49 ± 45 FJB-positive cells/ipsilateral hippocampus were counted; and at 4 wk after injury, no FJB-positive cells were detected. The FJB-positive cells were distributed throughout nearly the whole hippocampus from bregma −1.34 mm to bregma −4.04 mm (Fig. 3L). These results indicate that the cell death in the hippocampus of juvenile mice occurred almost simultaneously with the cell death in the cortex. The hippocampus also exhibited a sharp wave of cell death in a very short period between 24 h and 48 h after injury.

### TBI also induced neuronal death in the thalamus of juvenile mice

**Figure 4:**
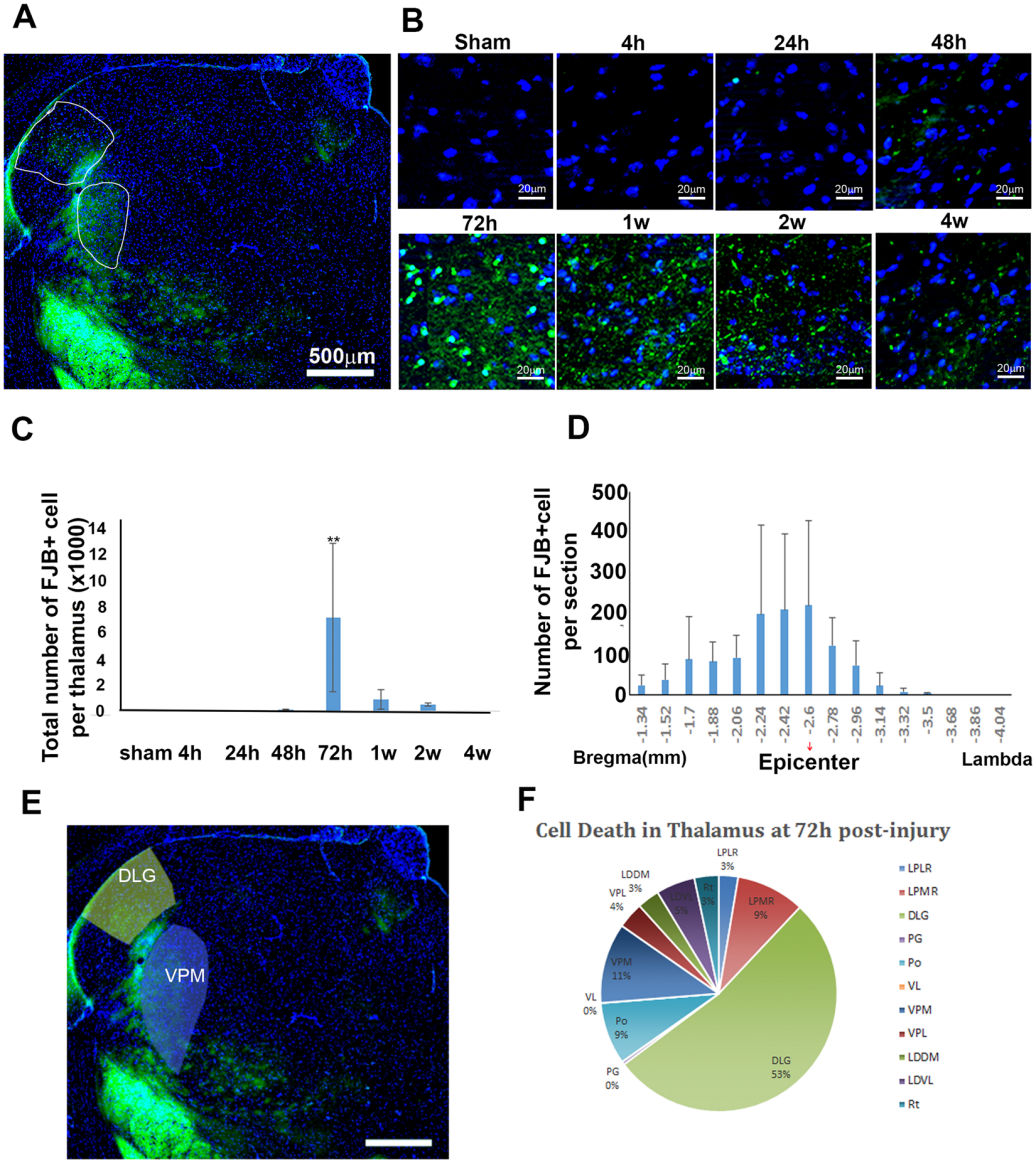
Temporal and spatial patterns of neuronal death in thalamus after TBI. (**A**) Fluoro-Jade B staining (green) showed the neuronal death in thalamus after injury. (**B**) The temporal pattern of neuronal death in thalamus after injury. (**C**) Quantification of FJB stained cells in thalamus (n = 4, **p < 0.01). (**D**) The rostral-caudal distribution of FJB-positive cells in ipsilateral thalamus at post-injury 72 h. (**E**) The regions where FJB-positive cells accumulated. (**F**) Quantification of FJB stained cells in different nuclei. LPMR: lateral posterior thalamic nucleus, mediorostral part; LPLR: lateral posterior thalamic nucleus, laterorostral part; DLG: dorsal lateral geniculate nucleus; PG: pregeniculate nucleus; Po: posterior thalamic nuclear group; VL: ventralateral thalamic nucleus; VPM: ventral posteromedial thalamic nucleus; VPL: ventral posterolateral thalamic nucleus; LDDM: laterodorsal thalamic nucleus, dorsalmedial part; LDVL: laterodorsal thalamic nucleus, ventrolateral part; Rt: reticular thalamic nucleus.

In addition to inducing dramatic neuronal death in the cortex and in the hippocampus both in the juvenile mice and young adult mice, moderate TBI also induced a much greater degree of neuronal death in the thalamus of juvenile mice than in the young adult mice, even though the thalamus is a relatively remote region from the impacted epicenter. Brain sections from multiple time points as described above were used to detect neuronal death in the thalamus with FJB staining. In the sham group no FJB-positive cells were observed in the thalamus (Fig. 4B). In the injured juvenile mice (Fig. 4B), no FJB-positive cells were observed at 4 h post-injury. At 24 h post-injury a few FJB-positive cells were seen in the thalamus (24 h: 14 ± 24 cells/ipsilateral thalamus, *p*_(24h vs. sham)_ = 0.994). The number of FJB-positive cells increased slightly from 24 h to 48 h after injury (48 h: 152 ± 50 cells/ipsilateral thalamus, *p*_(24h vs. 48h)_ = 0.941, *p*_(sham vs. 48h)_ = 0.931) (Fig. 4C). At 72 h post-injury, the number of FJB-positive cells in thalamus sharply increased compared to previous time points (72 h: 7146 ± 5941 cells/ipsilateral thalamus, *p*_(48h vs. 72h)_ = 0.001). Then the FJB-positive cells dramatically decreased and approached to sham level at 1 wk after injury (1 wk: 892 ± 750 cells/ipsilateral thalamus, *p*_(1w vs. 72h)_ = 0.002, *p*_(1w vs. sham)_ = 0.610). From 1 wk to 2 wk post-injury, the number of FJB stained cells decreased marginally from 892 ± 750 cells to 535 ± 128 cells in ipsilateral thalamus (*p*_(1w vs. 2w)_ = 0.838). At 4 wk after injury, only a few FJB stained cells were observed in ipsilateral thalamus (data not shown). The FJB-positive cells in juvenile thalamus start from bregma −1.34 mm and end at bregma −3.5 mm (Fig. 4D). Meanwhile, we found that the neuronal death in thalamus were not evenly distributed in the whole thalamus; they were more likely to accumulate in some nuclei (Fig. 4E). The quantification results showed that 53% of the FJB-positive cells were in the dorsal lateral geniculate nucleus, which received connections from the primary visual cortex^55^, suggesting that it may contribute to the visual issue in the pediatric patients. As well, there were 11% of FJB-positive cells within the ventral posteromedial thalamic nucleus, which is related to soma sensory function. The above results indicated that moderate TBI also triggered a delayed neuronal loss in the thalamus in the juvenile mouse brain, which is not commonly observed in the young adult mouse brain^43,47,51^.

### Neuronal death was not observed in more remote regions, such as hypothalamus and amygdala

We also observed more remote regions, such as the hypothalamus and the amygdala (Supplemental Figure 2A). FJB stained cells were not observed in hypothalamus of the sham group or any time point after injury (Supplemental Figure 2B). The same phenomenon was observed in the amygdala (Supplemental Figure 2C). The results indicated that moderate TBI rarely causes acute neuronal death in those remote regions in the juvenile mouse brain.

## Discussion

Although TBI is a serious medical issue, regardless of age, younger ages showing less resilience to the injury with more severe acute symptoms and more profound long-term deficits in cognition and sociability^8^. We aim to understand the mechanism using a rodent TBI model. Juvenile male mice at the age of 21 days received a moderate CCI injury, we then measured tissue lesion and neuronal death on their brains and compared them to our previous reports on brain injury in the young adult mice that received same level of impact using same injury device^43,47,51^. Although moderate CCI injury causes significant brain tissue lesion and cell death in in the ipsilateral cortex of both juvenile and young adult mice, the pattern of tissue lesion and neuronal death of juvenile mice is quite different from the pattern seen in young adult mice^51^. We found there are significant differences in neuropathologies between juvenile mice and young adult mice following moderate traumatic brain injury, including brain tissue lesion, tissue swelling, as well as neuronal death in the cortex, in the hippocampus, in the thalamus, and other regions in the brain. These differences in the pathologies may partially explain the differences in clinical observation between toddler and adult TBI patients, and may give suggestions to improve clinical cares for toddler TBI patients.

There are obvious differences in the characteristics of tissue lesion following TBI were seen between the juvenile mice and young adult mice^43,47,51^. In the juvenile mice, very little cavity due to tissue lesion was observed in the cortex at 4 h after TBI. The cavity in the cortex became obvious at 24 h after injury and continued to increasingly enlarge after 24 h and up to 2 wk. In contrast, a cavity in the cortex was easily observed at 4 h after injury in the young adult mouse brain, and the size of the cavity enlarged from 4 h to 72 h, but it did not continue expanding from 72 h after injury^51^. At 4 wk, the tissue lesion in the cortex of juvenile mice is 32.7% larger than in the young adult mice^51^. These data indicate that tissue lesion in the cortex was initially much less in the first few hours after injury in the juvenile mice than in the young adult mice. However, once the injury occurred, it developed more rapidly, lasted much longer, and eventually led to larger tissue lesion cavity in the cortex of juvenile mouse brain than of young adult mouse brain. The same level of injury causes a delayed but longer lasting tissue lesion, and a larger tissue lesion cavity in the cortex of juvenile mice than in the cortex of young adult mice.

Moderate TBI to juvenile and young adult mice induces both similar and different characteristics of neuronal death in the cortex at the same time. Moderate CCI injury induced a large amount of neuronal death in the ipsilateral cortex of both juvenile and young adult mice. Once neuronal death occurred, it came as a big wave of cell death that lasted a relatively short period of time, about 24 h in both juvenile and young adult mice. However, the huge wave of neuronal death occurred much earlier at 4 h after injury in the young adult brain, and occurred much later at 24 h after injury in the juvenile brain.

The neuronal death after injury in juvenile hippocampus was quite different from young adult hippocampus^51^. In young adult hippocampus, the number of FJB-positive cells dramatically increased at 4 h after injury (9479 ± 1828/ipsilateral hippocampus), and peaked at 24 h (12482 ± 1074/ipsilateral hippocampus). This peak value seen in young adult hippocampus was nearly 2-fold the number seen in juvenile hippocampus. From post-injury 48 h, FJB-positive cells reduced significantly and gradually declined until 2 wk. These results indicate that the cell death in the hippocampus of juvenile mice occurred almost simultaneously with the cell death in the cortex. The hippocampus also exhibited a sharp wave of cell death in a very short period between 24 h and 48 h after injury. Cell death in the hippocampus also was delayed in the juvenile mice compared with cell death in the hippocampus of young adult mice.

Moreover, we found significant cell death in the thalamus of juvenile brains at 72 h, which was not commonly seen in the young adult mice at this level of injury. In summary, cell death in juvenile mice was delayed, lasted longer, and finally resulted in more severe brain injury than in the young adult mice. This delayed cell death suggests there is a longer therapeutic time window for the juvenile mice. These results may partially explain why, upon the same impact, the symptoms are commonly more severe in the pediatric TBI patients than in the young adult TBI patients. These results also suggest that pediatric TBI patients may have a longer therapeutic window, but they also require longer intensive clinical care after injury than young adult TBI patients.

The mechanisms influencing the vulnerability of the young brain to TBI are poorly understood. Young people are at greater risk of severe brain injuries partially because their developing brains are undergoing a complex and sensitive process of growth. Humans are born with a brain about 28% of the adult weight. In toddlers the brain grows rapidly and reaches 90–95% of adult weight^56–59^. The human toddler brain is extraordinarily busy in growth and network formation. In toddlers, brain growth is at its highest rate, including dendrite development, axonal outgrowth, sprouting, synaptogenesis, myelination, and neurotransmitter and receptor changes^60^. Also in toddlers, the synapse density is at its peak compared with any other age, which is 50% higher than in the adult brain^61,62^. The myelination rate also peaks in the toddler brain^63^. There is a massive neural network formation, strengthening of cortical networks, and reorganization that occurs within the brain during this age. Furthermore, at this age the glial response^64^, neuroinflammation, and blood-brain barrier integrity may be differentially affected by TBI. Thus it is reasonable to hypothesize that the effects of TBI on a developing brain can be more detrimental. Furthermore, it will be interested to study whether a developing brain has more potential in neuroplasticity for self-repair follow TBI.

## Electronic supplementary material

supplemental data
