# Supplementary material for: Delayed and progressive damages to juvenile mice after moderate traumatic brain injury
Source: Sci Rep. 2018 May 9;8:7339. doi: 10.1038/s41598-018-25475-9 (PMC5943589; doi:10.1038/s41598-018-25475-9)
Supplement: Supplementary file 1 — supplemental data [file 41598_2018_25475_MOESM1_ESM.pdf]

Supplemental data

Delayed and progressive damages to juvenile mice after moderate traumatic brain injury

Shu Zhao[1], Xiaoting Wang[1], Xiang Gao[1], Jinhui Chen[1,2]

[1] Spinal Cord and Brain Injury Research Group, Stark Neuroscience Research Institute, Department of Neurosurgery, Indiana University, 320 W 15th street, Indianapolis, IN 46202

[2] **Corresponding author:**

Jinhui Chen MD & PhD
Neurosciences Research Building

320 W 15th Street
Indianapolis, IN 46202

Department of neurological Surgery and Stark Neuroscience Research Institute
Indiana University
Telephone: (317) 278-5782
Fax: (317) 278-5849
Email: chen204@iupui.edu

Supplemental figure 1

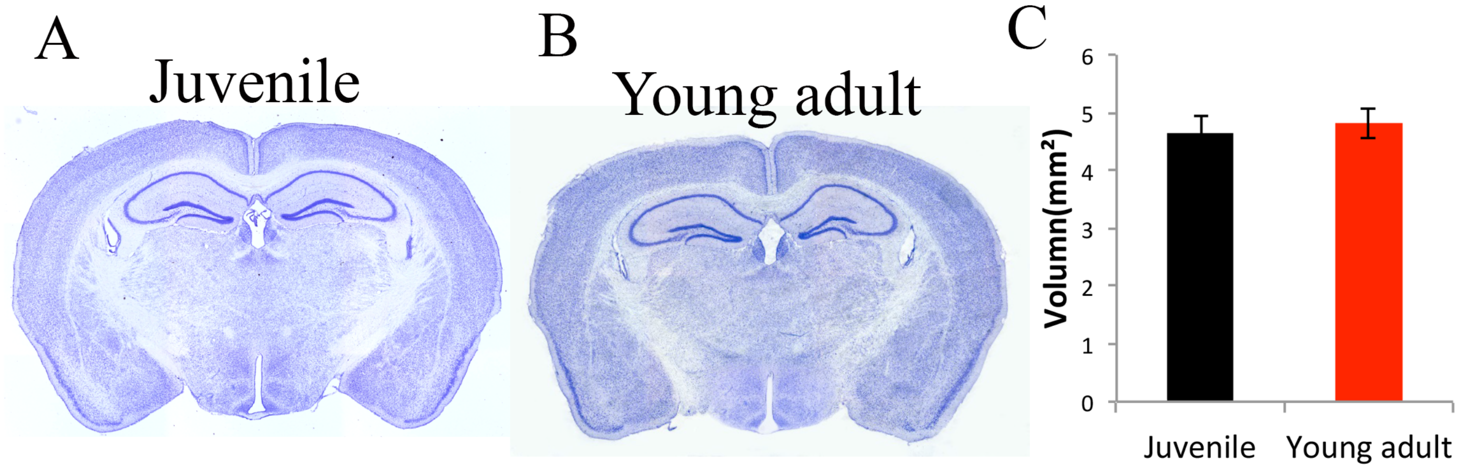

Suplemental figure 2

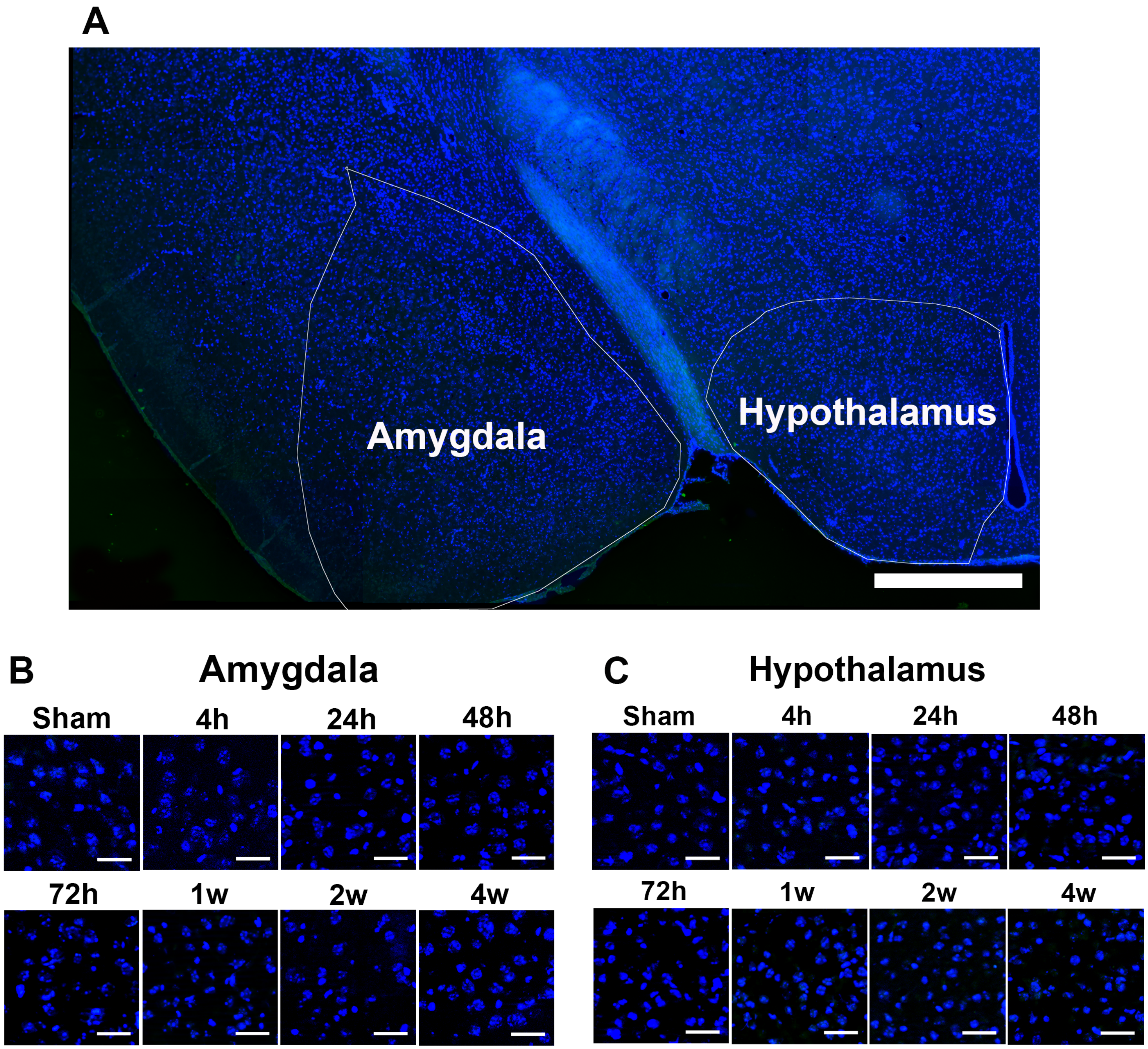

Supplemental figure legends

Supplemental figure 1. Comparing the cortex size between juvenile and young adult mice.

(A) Coronal section of juvenile mouse brain. (B) Coronal section of young adult mouse brain. (C). There is no significant difference in the cortex sizes between juvenile and young adult mice at this age.

Supplemental figure 2. No neuronal death was detected at amygdala and hypothalamus regions after TBI.

(A) Result of FJB staining in hypothalamus after injury. (B) Result of FJB staining in amygdala after injury.
